# Application of Sparse Autoencoders to Enhance Mechanistic Interpretability of Large Language Models in Medicine

**DOI:** 10.2196/81134

**Published:** 2026-05-27

**Authors:** Andre Metzger, Shiv Patil, Lauren R Sugarmann, Mert Karabacak, Konstantinos Margetis

**Affiliations:** 1 Mount Sinai Health System, 1468 Madison Avenue, New York, NY, United States, 1 212 241 3649; 2 Department of Neurosurgery, The Warren Alpert Medical School of Brown University, Providence, RI, United States

**Keywords:** large language model, artificial intelligence, sparse autoencoder, mechanistic interpretability, medical education

## Abstract

Large language models (LLMs) are being increasingly incorporated into clinical workflows due to their ability to synthesize medical knowledge and support diagnosis and treatment planning. However, their opaque internal decision-making processes limit trust, reliability, and safe clinical adoption. Mechanistic interpretability seeks to address this challenge by revealing how LLMs transform inputs into outputs. This paper explores the use of sparse autoencoders (SAEs) as a promising approach to improving mechanistic interpretability of LLMs in medicine. We discuss how SAE-based analyses can illuminate model reasoning, detect potential failure modes, and complement existing interpretability frameworks. Improving mechanistic interpretability through SAEs may be essential for safely deploying LLMs as trustworthy cognitive aids in clinical medicine.

## Introduction

The use of large language models (LLMs) in health care has substantially increased in recent years and is predicted to continue to grow [1,2]. While use of artificial intelligence (AI) in medicine may seem startling to some, its use in clinical practice is supported by LLM model performance on medical tasks such as answering US medical licensing–style questions with human-like accuracy [3,4]. When used as a supplement to standard clinical practice, AI-assisted care consistently outperformed human-only care [5,6]. These positive findings are encouraging for integrated use of LLMs and AI technology in clinical practice; however, alongside these findings, there is well-substantiated concern about LLMs generating false claims, increasing the risk for medical error [7,8]. There is currently no established mechanism to validate the decision-making process of LLMs, and this opacity creates a substantial barrier for developing the trust in AI that is required for complete adoption into clinical settings [9,10]. In order to safely incorporate LLMs in health care, both clinicians and AI subject-matter experts agree that these models must become more reliable. AI transparency can be improved by increasing mechanistic interpretability through the use of sparse autoencoders (SAEs) to track how external inputs influence LLM outputs. It is evident that use of LLMs in clinical care will continue to increase, and SAEs may offer a promising solution to understand algorithmic decision-making and safeguard patient care.

## Mechanistic Interpretability of LLMs

The goal of physician training is not simply to possess medical knowledge, but also to develop a nuanced reasoning process for examining evidence, understanding medical diagnoses, and issuing treatment plans [11]. Physicians spend years during and after medical school honing their thinking process in clinical rotations, residency, and fellowships. The development of LLMs should adopt such a framework if we aim to reliably integrate these tools into clinical workflows. Mechanistic interpretability is an emerging field of AI research seeking to unlock the “black box” of neural networks and reveal how these models generate decisions from internal computations [12]. In other words, rather than treating an LLM as an inscrutable oracle, mechanistic interpretability research is directed at dissecting the algorithms and representations that an LLM has learned in training to generate its output. Recent developments highlight the potential value of this approach.

It is important to note that current mechanistic interpretability tools, including SAEs, provide feature-level decompositions of model activations at specific layers rather than a comprehensive deconstruction of the model’s end-to-end thought process. These SAEs must be trained individually for every layer of interest of a model to provide a window into the intermediate representation at that layer. This makes SAEs useful tools to identify which learned concepts the model is prioritizing at the stage of processing and to form hypotheses about the model’s decision-making process [13].

Researchers have begun to map out how models process, contextualize, and generate information in foundation models such as OpenAI’s GPT or Anthropic’s Claude [14]. Such findings illustrate the potential of identifying cause-and-effect explanations for specific model behaviors. If we extend this to clinical applications, we may understand how an LLM weighs symptoms and risk factors to inform its reasoning when suggesting a potential diagnosis. This deeper understanding could help us detect aberrant systematic processes the model may have and correct these issues before a medical error occurs. For instance, if an LLM erroneously recommends an unsafe medication, a mechanistic interpretability analysis might reveal whether the model ignored a drug allergy in the prompt or if a spurious association in its training data led to this mistake. Knowing why the model failed may allow us to prevent similar errors in the future and allow us to institute safeguards in deployment. It is possible, however, that some of this failure may be rooted in the way that AI models are trained and the goals that LLMs have been optimized to achieve.

Early evaluations of LLMs in medicine often focused on board-style exams and quizzes, demonstrating that LLMs can accurately recall and apply a wide range of medical knowledge. Yet, correctly answering multiple-choice questions has little use in true clinical practice. In recognition of this discrepancy, recent benchmark studies have designed more nuanced evaluations of LLM learning that have revealed significant shortcomings. For example, when LLMs were presented with authentic patient case data from an intensive care unit and asked to triage, diagnose, and recommend treatments to patients in a simulated clinical workflow, advanced LLMs consistently missed diagnoses, failed to follow established diagnostic and treatment guidelines, and often could not interpret laboratory data trends [15]. Use of mechanistic interpretability may enable a clearer understanding of the existing limitations of these models.

## Sparse Autoencoders

**Figure 1.**
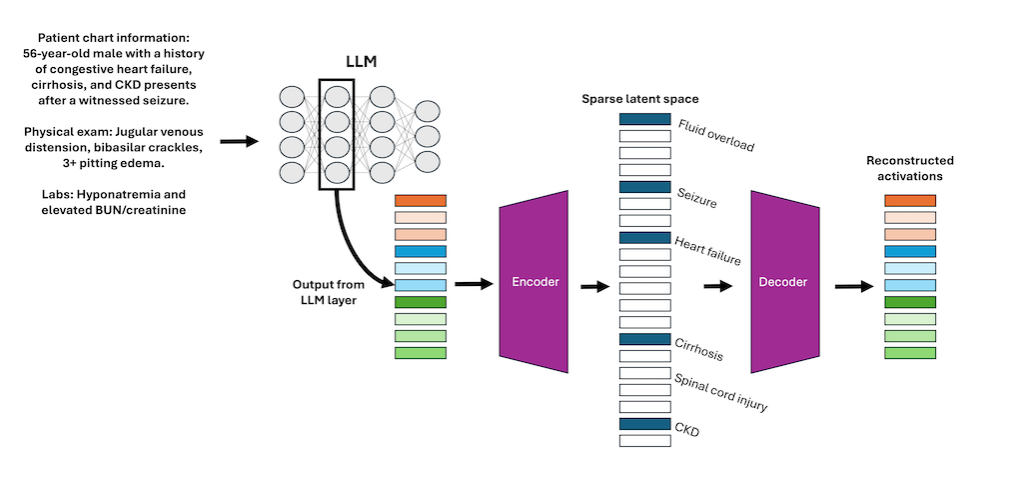
Integration of a sparse autoencoder (SAE) within a large language model (LLM) to improve mechanistic interpretability in a hypothetical clinical scenario. Information from a patient chart is provided as input to the LLM. Isolating a hidden layer of the LLM, an SAE transforms polysemantic activations from this layer into monosemantic vectors that can be associated with individual, human-interpretable features. The SAE implements a sparsity penalty during training to reward the model for activating fewer nodes in the model, thereby ensuring greater specificity of such nodes. BUN: blood urea nitrogen; CKD: chronic kidney disease.

One approach to enhancing LLM mechanistic interpretability in medicine involves the use of algorithms called SAEs (Figure 1). SAEs are a subclass of the standard autoencoder architecture, a class of neural network trained to learn a representation (encoding) of its input in a dimensionally different space (latent space) from the original input space and subsequently reconstruct the original input from the compressed representation [16]. A standard autoencoder consists of two primary components, an encoder function that maps the input (*x*) to a latent encoding (*c*) and a decoder function that maps the latent encoding back into the original input space to create a reconstruction of the input ($\hat{x}$). The autoencoder networks are trained to minimize the reconstruction error between the input *x* and the autoencoder’s reconstruction, $\hat{x}$.

**Figure 2.**
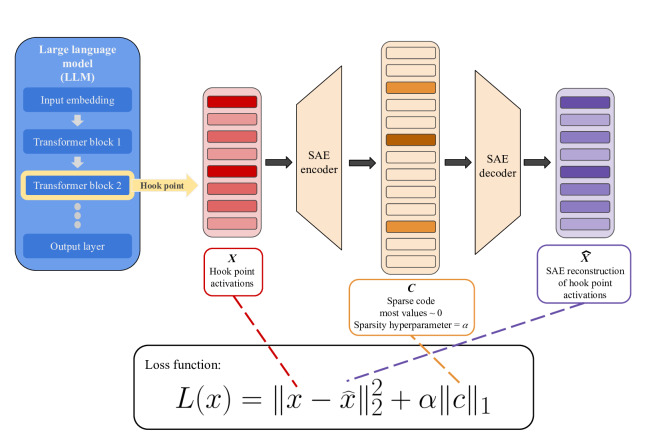
SAE training pipeline. Intermediate activations, *X*, from a layer of a large language model are passed through an encoder to produce a sparse latent code, *c*, which is then decoded to make a reconstruction of the original activations, $\hat{X}$. The SAE is trained using a loss function that minimizes reconstruction error while applying an L1 penalty to the latent code, encouraging sparsity and promoting more interpretable feature representations. The sparsity hyperparameter, α, is a predetermined scalar coefficient, typically tuned via hyperparameter search. SAE: sparse autoencoder.

Traditionally, autoencoders have been used to learn compressed, lower-dimensional representations of data while preserving essential information, with applications in dimensionality reduction, feature learning, and denoising across domains [16]. SAEs are an extension of this framework that operate in a latent space that is higher-dimensional than the input space and introduce a sparsity constraint on the latent representation. This constraint encourages a small number of latent features to have high activations while the majority of latent features are near zero (Figure 2). As a result of adding the sparsity constraint, the model is trained for the most important latent features to be activated. This promotes the emergence of distinct, interpretable features, enabling individual latent units to be associated with specific interpretable concepts [13,17].

When given an input, an LLM encodes information into a multidimensional array of numbers. Each number, or node, is influenced by the complete input and is not a monosemantic representation of a single word or concept. Accordingly, the information for a single word or concept from the input, such as “heart failure” or “anxiety,” is spread across multiple nodes [13,16-18]. This phenomenon, known as superposition, is where individual neurons do not correspond to single, identifiable concepts, but instead, many concepts are encoded simultaneously across overlapping sets of neurons rather than being localized to single interpretable units. SAEs can address this by learning to decompose these dense, superimposed activations into a higher-dimensional latent space where individual features are more likely to correspond to monosemantic, human-interpretable concepts [17]. As inputs pass through each layer of the model, different computations are performed that iteratively modify the representations until a final output is produced. In this context, SAEs can serve as a post hoc analysis tool to a trained LLM to identify the most important features in the model’s current representation.

An SAE is trained on a hook point, an internal activation at a specific layer of the LLM. At this chosen point in the LLM’s computations, a callback mechanism intercepts and records the intermediate activation vector as it passes through the model. Capturing this vector enables it to be used for analysis by the SAE without altering the LLM’s computations or outputs. The SAE takes this representation and translates it into an expanded latent space that effectively constructs a larger “dictionary” of features, some of which correspond to identifiable monosemantic concepts. By expanding the hook point representation into this higher-dimensional space, researchers can identify the features a model uses to represent information, providing insight into how the model processes data [19].

In transformer-based LLMs, SAEs are most commonly trained on the post multilayer perceptron residual stream, which contains the model’s updated representation after the feed-forward network in a transformer block [19,20]. However, other locations may also be used depending on the interpretability goal, including multilayer perceptron outputs after layer normalization or the combined outputs of multiple attention heads before the model projects them back into the residual stream [19-21].

The SAE places constraints that diminish widespread latent activation, encouraging the model to represent each input using a small subset of activated features. The most common SAEs, local reconstruction SAEs, aim to create a sparse code (*c*), a representation of the inputted hook point activation (*x*), in the latent space. The SAEs then aim to reconstruct the hook point vector from the sparse code. This is done by optimizing the SAEs’ loss function: $L\left(x\right)=||x-\hat{x}|{|}_{2}^{2}+\alpha ||c|{|}_{1}$. This loss function penalizes the difference between the inputted hook point activation (*x*) and the SAEs’ reconstruction of the input ($\hat{x}$), as well as the sparsity of the latent code (*c*), where α is the sparsity hyperparameter that controls the trade-off between accurate reconstruction and the degree of sparsity imposed on the latent code (Figure 2) [17,19]. However, sparsity can be enforced through different mechanisms. While this classical approach applies an L1 penalty to encourage only a few latent features to activate, other loss functions such as TopK SAEs restrict the representation to the *k* strongest features. More recent approaches, such as JumpReLU SAEs, introduce learnable activation thresholds that more directly control how many features are active at once. In addition to these reconstruction-based approaches, newer methods train SAEs end-to-end by optimizing the model so that replacing the original internal representation with the reconstructed version preserves the model’s output distribution. This approach prioritizes features that maintain the model’s functional behavior rather than simply reproducing the original activations [19,20,22].

Selecting the number of latent features and the level of sparsity is also an important design choice when training SAEs. Rather than relying on a single configuration, researchers typically train multiple models with different dictionary widths and sparsity levels to evaluate them using several criteria. These criteria often include how well the reconstructed activations preserve the model’s prediction behavior, the number or proportion of features that never activate, “dead latents,” automated interpretability scores, and measures of how well individual features represent distinct concepts [19].

When trained and applied to an LLM, an SAE can help identify what part of the information the model is prioritizing at a chosen layer. In the training process, monosemantic concepts are identified by requiring the associated node to activate across a minimum number of inputs and examining the types of text or clinical features that trigger high activation of the feature. Through this process, SAEs can reveal interpretable internal features that represent a clinical concept, trend, or linguistic pattern used by the model when generating outputs [19,20]. This identification can be applied to help improve the mechanistic interpretability of LLMs used for patient care. For example, if a model is tasked with providing a recommendation for treatment of a patient with an acute heart failure exacerbation, SAE-derived features for “interstitial edema,” “cardiomegaly on chest x-ray,” “congestive heart failure,” or “Xarelto” may be given to contextualize the LLM’s output of acute heart failure [13,17-19,23]. This insight can be useful for understanding the process and key information a model is using as well as to check its processing. The features independently may raise suspicion for an episode of acute decompensated heart failure exacerbation; they do not confirm it. If these features also co-activate with a node associated with “elevated NT-proBNP” and “ultrasound feature of reduced ejection fraction,” the output can become more meaningful. In this function, SAEs should be understood as post hoc auditing tools rather than clinical validation tools. SAE-derived features can contextualize a model’s output, identify clinically relevant or concerning internal representations, and generate hypotheses about possible failure modes, but they do not independently establish that the model’s recommendation is correct or safe [24,25].

Analyzing adjacent layers can also provide insight into the sequential decision-making process. Evaluating what the model identifies as the most important features can reveal potential explanatory factors the model identifies within large datasets that would otherwise require time-intensive chart review to extract [26]. In clinical deployment, SAE outputs can be used as additional explanation via visualization of the most important features at various stages of a model’s process visualized in a flowchart or in a natural language explanation along with where this data came from and any associated uncertainty indicator. This application of SAEs can be integrated within clinical decision support tools that live in the electronic health record or as standalone interfaces designed to foster trust through deep clinician auditing of a model’s information processing. Another application of SAEs in medicine that aligns with their historical use in computer vision is encoding high-dimensional image-derived data such as chest X-rays, mammograms, digital pathology whole-slide images, echocardiography videos, angiography images, and computed tomography volumes into monosemantic descriptions of the image that is more amenable to integration with LLMs trained on text reports [27-35].

## SAE vs Other Interpretability Frameworks

SAEs stand out against other interpretability methods of medical LLMs for their ability to reveal and potentially intervene in the internal features and decision-making process of the model. Local interpretable model-agnostic explanations (LIME), Shapley additive explanations (SHAP), and gradient-based interpretability methods identify key input variables by assessing changes in outputs when inputs are systematically modified. While these methods can be useful to ensure that key information is used and spurious information is not prioritized, they offer limited insight and no actionable modification to the model’s decision-making process [36-38]. SHAP and LIME methods require extensive computational expense, as they run the model with each change in input, whereas gradient-based methods rely on making changes to the internal computations of the model. Other methods, such as attention-based methods, rely on running multiple inputs and correlating the inputs to nodes that see increased activation. Attention-based interpretability has known faithfulness problems and fails to provide interpretable explanations of what the highlighted nodes represent [39]. In a recent medical-coding study, label attention highlighted extraneous tokens due to polysemanticity, while sparse autoencoders provided more mechanistically grounded explanations [40]. Recent attention to chain-of-thought–based prompting has emerged to provide physicians with the model’s account of its decision-making process. However, this approach does not provide reliable insight into what the model truly considered when making a diagnosis but rather what the model sees as the most fitting explanation [41]. In contrast, SAEs afford insight into the model at any layer rather than abstracting causality from input-output relationships. Applying SAEs across multiple layers may help trace how clinically relevant feature activations evolve through the model, although this should not be interpreted as faithful reconstruction of the model’s reasoning process.

## Challenges and Limitations

A fundamental challenge of SAE evaluation and implementation is ensuring the extraction of identifiable and human-recognizable features. Current evaluation methods rely on subject matter experts to identify the monosemantic representations of latent-space nodes, meaning that physicians must be hired to characterize nodes in response to medical terms or laboratory values [41]. Because human-dependent processes are inherently subjective, different nodes may be labeled differently by each reviewer, meaning that each SAE may be inherently different. This subjectivity is commonly referred to as the “ground truth paradox” [42]. For example, physicians hired to label a monosemantic node that activates when “chest pain,” “ST segment,” and “troponin” are inputted to the model may provide different responses such as “myocardial infarction” or “unstable angina.” Not only does requiring physician responses introduce variability into SAEs, but this process is also expensive and labor intensive. While fully developed models may reduce physician workload in the future, developing functional SAEs in this manner is a time-intensive process and may increase short-term physician workload. This process is also costly, both environmentally and monetarily. Training SAEs on large language model activations can require substantial computational resources. For example, SAEs trained on tens of billions of activation tokens typically require 500‐3000 GPU hours depending on the model size, dictionary width, and sparsity constraints [19,43]. Using commonly used accelerators such as NVIDIA A100 GPUs, which draw 300‐400 W under training workloads, a 1000 GPU-hour training run consumes 300‐400 kWh of electricity. Assuming average US electricity carbon intensity (~0.4 kg CO₂ per kWh), this corresponds to 120‐160 kg of CO₂ emissions, while larger training runs approaching 3000 GPU-hours may emit 350‐500 kg of CO₂ [44,45]. The financial costs are similarly nontrivial. At typical cloud pricing as of March 2026, at $2-$4 per A100 GPU hour, training a single SAE model may cost $1000-$4000, with larger experimental sweeps involving multiple sparsity levels or dictionary sizes easily exceeding $10,000 in total compute costs [46,47]. These estimates highlight that even relatively modest SAE experiments require meaningful computational investment, while large-scale interpretability efforts analyzing many layers or models can scale far beyond these costs.

Thus, it is important that the development of medical SAEs be done across medical institutions such that the burden of labor, cost, and computation can be shared. This will also allow for greater diversity in physician perspectives to achieve a more reliable ground truth of the models. Multi-institution collaboration will be necessary to capture diversity in physician perspectives, and structured strategies should be used to reduce subjectivity. To mitigate the ground-truth paradox, datasets should preserve multirater label distributions, report interrater agreement, perform calibration rounds using standardized annotation rubrics, require rationale capture to enhance learnability, and use senior adjudication only for persistently ambiguous examples. Rather than defaulting to simple majority vote, consensus should be weighted by an annotator’s consistency and learnability of their rationale. Models should also be trained with uncertainty-aware objectives so that disagreement is represented explicitly and can influence physicians’ interpretation of results. Finally, postdeployment monitoring must be performed and should include label-free reliability audits on out-of-distribution clinical data [8,42,48,49]. Another limitation to SAE use in health care is their representation of diseases in a monosemantic latent space. While SAEs in health care LLMs may provide clear monosemantic nodes for a specific patient presentation, not all patients present the same way, and various conditions present in the same way. Similarly, patients often have multiple morbidities contributing to their presentation.

## Future Directions

Research on SAEs and LLM use in medicine is ongoing, and there are several applications of SAEs that warrant further investigation and development. The insight into a model’s decision-making process can enable the development of process-oriented benchmarks to ensure a model’s reasoning steps align with what is expected in clinical reasoning. Just as medical educators evaluate how a student worked through a case (not only whether they got the correct answer), SAEs could allow us to evaluate how a model arrived at its conclusion. This is particularly relevant as the field of medical AI is seeking to depart from medical assessment–based validations [50,51]. As recent studies have called for increased interpretability for LLMs to address shortcomings when transitioning from structured diagnostic questions to complex subspecialty scenarios, SAEs warrant exploration as a potential mechanism to provide this deeper insight into models similar to how interpretability methods like Grad-CAM have increased the interpretability, performance, and trust in computer-vision assisted radiology [52-54].

Another area of future consideration is the use of SAEs to guardrail agentic decision-making by developing a way to modify how a model weighs information during its processing. This application of SAEs should be explored to improve a model’s guideline adherence when making decisions. This application of SAEs can also be expanded to explore the implications of allowing physicians to input their own preferences for how an algorithm is approaching a case by placing their own guidelines or preferences in monosemantic category amplification during the decision-making process. Developing a model with this capability would allow physicians to have more explicit control and ownership over the model’s decision-making process than unreliably prompting the agent to follow a guideline or telling it how to weigh clinical signals in a prompt. The implementation of SAEs in clinical workflows also requires further exploration. The application and depth of an SAE’s analysis of an LLM decision-making process can range from briefly supplementing decision support tool outputs embedded in clinical workflow to enabling detailed auditing of a model focused on building trust. Both avenues should be explored to develop a toolkit not only to enhance AI as a supportive tool for clinicians but to foster trust in the tools at their disposal. As SAEs develop, physicians should be deeply involved in how products are designed to fit into electronic health records and provide insight into how clinical decision support tools are processing information. Future research is also needed regarding ethical considerations that may arise when uncovering how an LLM “thinks,” such as the potential to learn that an LLM has used protected attributes such as race, gender, or socioeconomic status. Future research on how to address these concerns must be conducted.

## Conclusion

The integration of LLM use into clinical care will only continue to grow in the coming years. SAEs offer a promising solution to mitigate model error and expand “trust” in LLM outputs by improving mechanistic interpretability. Ultimately, understanding the internal processes of LLMs may be key to defining their role in clinical workflows and addressing potential ethical concerns that may be identified through SAE analysis. SAEs can illuminate where these models excel, where they are brittle, and how they might be safely integrated as cognitive aids for health care professionals, rather than being unreliable black boxes.

## Abbreviations

AI: artificial intelligence
LIME: local interpretable model-agnostic explanations
LLM: large language model
SAE: sparse autoencoder
SHAP: Shapley additive explanations

## References

[1] Allen B, Agarwal S, Coombs L, Wald C, Dreyer K (2021). 2020 ACR Data Science Institute artificial intelligence survey. J Am Coll Radiol.

[2] Sahni NR, Carrus B (2023). Artificial intelligence in U.S. health care delivery. N Engl J Med.

[3] Kung TH, Cheatham M, Medenilla A (2023). Performance of ChatGPT on USMLE: potential for AI-assisted medical education using large language models. PLOS Digit Health.

[4] Singhal K, Azizi S, Tu T (2023). Large language models encode clinical knowledge. Nature New Biol.

[5] Han R, Acosta JN, Shakeri Z, Ioannidis JPA, Topol EJ, Rajpurkar P (2024). Randomised controlled trials evaluating artificial intelligence in clinical practice: a scoping review. Lancet Digit Health.

[6] Lam TYT, Cheung MFK, Munro YL, Lim KM, Shung D, Sung JJY (2022). Randomized controlled trials of artificial intelligence in clinical practice: systematic review. J Med Internet Res.

[7] McDuff D, Schaekermann M, Tu T (2025). Towards accurate differential diagnosis with large language models. Nature New Biol.

[8] Asgari E, Montaña-Brown N, Dubois M (2025). A framework to assess clinical safety and hallucination rates of LLMs for medical text summarisation. NPJ Digit Med.

[9] Tang L, Sun Z, Idnay B (2023). Evaluating large language models on medical evidence summarization. NPJ Digit Med.

[10] Shah SV (2024). Accuracy, consistency, and hallucination of large language models when analyzing unstructured clinical notes in electronic medical records. JAMA Netw Open.

[11] Clusmann J, Kolbinger FR, Muti HS (2023). The future landscape of large language models in medicine. Commun Med (Lond).

[12] Williams CYK, Miao BY, Kornblith AE, Butte AJ (2024). Evaluating the use of large language models to provide clinical recommendations in the Emergency Department. Nat Commun.

[13] Chanin D, Wilken-Smith J, Dulka T, Bhatnagar H, Golechha S, Bloom J (2024). A is for absorption: studying feature splitting and absorption in sparse autoencoders. arXiv.

[14] Goh E, Gallo R, Hom J (2024). Large language model influence on diagnostic reasoning: a randomized clinical trial. JAMA Netw Open.

[15] Choudhury A, Chaudhry Z (2024). Large language models and user trust: consequence of self-referential learning loop and the deskilling of health care professionals. J Med Internet Res.

[16] Hinton GE, Salakhutdinov RR (2006). Reducing the dimensionality of data with neural networks. Science.

[17] Cunningham H, Ewart A, Riggs L, Huben R, Sharkey L (2023). Sparse autoencoders find highly interpretable features in language models. arXiv.

[18] Pach M, Karthik S, Bouniot Q, Belongie S, Akata Z (2025). Sparse autoencoders learn monosemantic features in vision-language models. arXiv.

[19] Gao L, la TT, Tillman H (2024). Scaling and evaluating sparse autoencoders. arXiv.

[20] Lawson T, Farnik L, Houghton C, Aitchison L (2024). Residual stream analysis with multi-layer SAEs. arXiv.

[21] Kissane C, Krzyzanowski R, Bloom JI, Conmy A, Nanda N (2024). Interpreting attention layer outputs with sparse autoencoders. arXiv.

[22] Rajamanoharan S, Lieberum T, Sonnerat N (2024). Jumping ahead: improving reconstruction fidelity with JumpReLU sparse autoencoders. arXiv.

[23] Shi W, Li S, Liang T (2025). Route sparse autoencoder to interpret large language models.

[24] Frasca M, La Torre D, Pravettoni G, Cutica I (2024). Explainable and interpretable artificial intelligence in medicine: a systematic bibliometric review. Discov Artif Intell.

[25] Scarpato N, Nourbakhsh A, Ferroni P (2024). Evaluating explainable machine learning models for clinicians. Cogn Comput.

[26] Olshausen BA, Field DJ (1997). Sparse coding with an overcomplete basis set: a strategy employed by V1?. Vision Res.

[27] Lyu D, Wang X, Chen Y, Wang F (2024). Language model and its interpretability in biomedicine: a scoping review. iScience.

[28] Abdulaal A, Fry H, Montaña-Brown N (2024). An x-ray is worth 15 features: sparse autoencoders for interpretable radiology report generation. arXiv.

[29] Nakka KK (2025). Mammo-SAE: interpreting breast cancer concept learning with sparse autoencoders. arXiv.

[30] Ding T, Wagner SJ, Song AH (2024). Multimodal whole slide foundation model for pathology. arXiv.

[31] Ahmed F, Sellergren A, Yang L (2024). PathAlign: a vision-language model for whole slide images in histopathology. arXiv.

[32] Vukadinovic M, Tang X, Yuan N (2024). EchoPrime: a multi-video view-informed vision-language model for comprehensive echocardiography interpretation. arXiv.

[33] Chen X, Zhang W, Xu P (2024). FFA-GPT: an automated pipeline for fundus fluorescein angiography interpretation and question-answer. NPJ Digit Med.

[34] Blankemeier L, Kumar A, Cohen JP (2026). Merlin: a computed tomography vision-language foundation model and dataset. Nature New Biol.

[35] Yang S, Du J, Guo J (2024). ViLReF: a chinese vision-language retinal foundation model. arXiv.

[36] Lundberg S, Lee SI (2017). A unified approach to interpreting model predictions. arXiv.

[37] Ribeiro MT, Singh S, Guestrin C (2016). "Why should I trust you?": Explaining the predictions of any classifier. arXiv.

[38] Widdicombe A, Julier SJ (2021). Gradient-based interpretability methods and binarized neural networks. arXiv.

[39] Jain S, Wallace BC (2019). Attention is not explanation. arXiv.

[40] Wu J, Wu D, Sun J (2025). Beyond label attention: transparency in language models for automated medical coding via dictionary learning. arXiv.

[41] Sinha A, Umakanthan OC, Gajendran S (2025). DR-CoT: dynamic recursive chain of thought with meta reasoning for parameter efficient models. Sci Rep.

[42] Sylolypavan A, Sleeman D, Wu H, Sim M (2023). The impact of inconsistent human annotations on AI driven clinical decision making. NPJ Digit Med.

[43] Lieberum T, Rajamanoharan S, Conmy A (2024). Gemma Scope: open sparse autoencoders everywhere all at once on Gemma 2. arXiv.

[44] Strubell E, Ganesh A, McCallum A (2019). Energy and policy considerations for deep learning in NLP. arXiv.

[45] Patterson D, Gonzalez J, Le Q (2021). Carbon emissions and large neural network training. arXiv.

[46] Low-cost GPU cloud services for AI startups in 2025. GMI Cloud.

[47] A100 GPU cloud comparison: pricing, performance & top providers. Runpod.

[48] Gao Z, Wittrup E, Najarian K (2024). Leveraging multi-annotator label uncertainties as privileged information for acute respiratory distress syndrome detection in chest X-ray images. Bioengineering (Basel).

[49] Kiyasseh D, Cohen A, Jiang C, Altieri N (2024). A framework for evaluating clinical artificial intelligence systems without ground-truth annotations. Nat Commun.

[50] Raji ID, Daneshjou R, Alsentzer E (2025). It’s time to bench the medical exam benchmark. NEJM AI.

[51] Azad TD, Krumholz HM, Saria S (2026). Principles to guide clinical AI readiness and move from benchmarks to real-world evaluation. Nat Med.

[52] Jagarapu J, Babata K, Chamarthi S, Hoyt R (2025). The accuracy and repeatability of openevidence on complex medical subspecialty scenarios: a pilot study. Health Informatics.

[53] Abbas Q, Jeong W, Lee SW (2025). Explainable AI in clinical decision support systems: a meta-analysis of methods, applications, and usability challenges. Healthcare (Basel).

[54] Chen E, Saenz A, Banerjee O (2025). International retrospective observational study of continual learning for AI on endotracheal tube placement from chest radiographs. NEJM AI.

